# A Semantic Model to Study Neural Organization of Language in Bilingualism

**DOI:** 10.1155/2010/350269

**Published:** 2010-03-01

**Authors:** M. Ursino, C. Cuppini, E. Magosso

**Affiliations:** Department of Electronics, Computer Science and Systems, University of Bologna, Viale Risorgimento 2, I40136 Bologna, Italy

## Abstract

A neural network model of object semantic representation is used to simulate learning of new words from a foreign language. The network consists of feature areas, devoted to description of object properties, and a lexical area, devoted to words representation. Neurons in the feature areas are implemented as Wilson-Cowan oscillators, to allow segmentation of different simultaneous objects via gamma-band synchronization. Excitatory synapses among neurons in the feature and lexical areas are learned, during a training phase, via a Hebbian rule. In this work, we first assume that some words in the first language (L1) and the corresponding object representations are initially learned during a preliminary training phase. Subsequently, second-language (L2) words are learned by simultaneously presenting the new word together with the L1 one. A competitive mechanism between the two words is also implemented by the use of inhibitory interneurons. Simulations show that, after a weak training, the L2 word allows retrieval of the object properties but requires engagement of the first language. Conversely, after a prolonged training, the L2 word becomes able to retrieve object per se. In this case, a conflict between words can occur, requiring a higher-level decision mechanism.

## 1. Introduction

The term semantic memory is commonly used to denote a kind of declarative memory which is independent of the context as well as culturally shared and involves words and concepts. Several theories of semantic memory have been developed in the past decades, with the aim of understanding how words are linked with object representation, and how this link is altered in pathological subjects with neurological deficits. In most of these theories, semantic memory is considered a distributed process, which involves many different cortical areas and adopts a multimodal (sensory-motor) representation of objects [1–4]. More specifically, in these theories an object is usually represented as a collection of features spreading across different sensory and motor modalities, which must be linked together and with the corresponding words. Hence, retrieval of objects from memory requires that all these distributed representations, and the corresponding words, be activated all together starting from sensory or lexical cues, and integrated to form a single coherent percept. Synchronization in the gamma band is nowadays assumed to play an essential role in high-level cognitive processes. Recent results suggest that gamma rhythms are involved in high-level object memorization and retrieval [5], and in linking words with senses [6]. 

Although the previous ideas are largely debated in the present neurocognitive literature, just a few mathematical models have been presented until now. Recently, we developed a mathematical model of object representation, in which abstract objects are described as a collection of features. In the model, features of the same object are linked together, and separated from those of different objects, via synchronization of neural oscillators in the gamma band. The network was able to recognize objects, and separate them from other objects simultaneously present, even in case of partial or corrupted information, and when objects share some common features [7, 8]. In a more recent version of the same model, this object representation, spreading across different feature areas, is linked with a lexical area devoted to word representation, so that correct object retrieval can evoke the corresponding word, and vice versa. Some simple “semantic” relationships between words which share common features were also realized with this network [9].

In the present work, the same “semantic” network is used to illustrate, with a simple exemplum, how the model can be used to describe the process of word learning in a second language. To this end, we assume that the network, previously trained with a few words in a first language (L1) (so that words are associated with object representation), learns a second-language (L2) word. The L2 word is associated with a previous L1 word via a Hebbian learning procedure. A competitive mechanism between words representing the same object is also implemented. After a prolonged training, the L2 word becomes able to retrieve the same object representation as the original word. The results are commented from the viewpoint of present hypotheses on second-language representation and control.

## 2. Method

The model consists of two different layers: the first (named “feature network”) is devoted to a description of objects represented as a collection of sensory-motor features. The second (named “lexical network”) is devoted to the representation of words, from an upstream process of phonemes. The two networks communicate via trained synapses. Moreover, the lexical network also receives a signal from a “decision network”, which recognizes whether a correct object information is present in the feature network and avoids that a misleading representation can evoke a word. A more complete description of the model, with equations and parameter values, can be found in previous works [8, 9]. 

In order to simulate learning of a second language, in the present model we included an additional mechanism not used before: two words, which represent the same object (the first, named L1 word, in the original language and the second, named L2 word, in the new language), interact via a competitive mechanism. This competition is realized by means of inhibitory interneurons. The synapses which originate from these interneurons are also subject to Hebbian learning 

**Figure 1:**
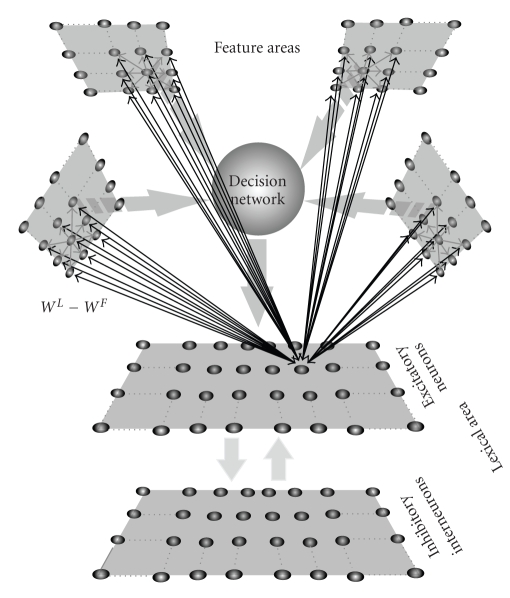
Schematic diagram describing the general structure of the network. The upper shadow squares represent distinct Feature Areas and are organized in a topological way. Each area embodies 20 × 20 elements (black circles), represented by means of Wilson-Cowan oscillators, devoted to the representation of a specific feature of an object. The lower squares represent the two populations of neurons in the Lexical Area: excitatory neurons, responsible for the representation of words, and inhibitory interneurons, involved in tasks like problem solving. Each of these areas is made of 40 × 40 elements (black circles), which are represented by a first-order dynamic and a sigmoidal relationship. Between the Feature and the Lexical Areas there is a decision network, which un-inhibits the lexical area, in case of correctly segmented objects. The model includes intra-area (excitatory and inhibitory) synapses among elements belonging to the same Feature Area, long-range excitatory interarea synapses between oscillators in different Feature Areas, and long-range excitatory synapses between elements in the Feature Network and in the Lexical Areas (*W* ^*F*^ − *W* ^*L*^).

A schematic description of model structure is presented in Figure 1.

### 2.1. The Feature Network

Each area in the feature network is devoted to the representation of a specific attribute or feature of the object, according to a topological organization. Hence, one object is represented as the collection of *F* features (one feature per each area). In this work we used *F* = 4. We assume that each attribute has been extracted from a previous processing in the neocortex, which elaborates sensory-motor information. Each unit in the feature areas consists of Wilson-Cowan oscillators [10, 11]. Oscillators in the same area are connected via lateral excitatory and inhibitory synapses, according to a classical “Mexican hat” disposition, which implements a “similarity principle”; this means that elements which signal similar attributes are located in proximal positions in the network; hence, they tend to be reciprocally connected and activate together. Neural oscillators belonging to different areas can be connected via excitatory synapses after training. These synapses are initially set to zero, but may assume a positive value through a learning phase, to memorize “prior knowledge” on attributes occurring together during the presentation of objects. Lateral synapses are not subjected to a training phase. All equations can be found in [7–9].

### 2.2. Lexical Area

Each unit represents a specific “word”. It can receive an input from a preprocessing stage which detects words from phonemes but it can also be stimulated through long-range synapses coming from the feature network; in this way, a “word” is linked with elements in feature areas representing specific properties of a stored object. All together, a “word” and its specific attributes are combined to embody the semantic meaning of that concept and the integrated network can indifferently be activated by language or sensory-motor information of an object.

In the present model we assume that the lexical network can be activated by the elements of the feature areas only if a “decision network” is in the on state. This is realized sending sufficient inhibition to all elements of the lexical area. This inhibition is withdrawn by the decision network, as soon as an object is recognized.

This network is located downstream of the Feature Network. It receives input from all the elements of the Feature Areas and verifies that (1) there is an “activation bubble” in any area. (2) Any area produces just a single activation bubble at a given instant. (3) The conditions (1) and (2) are verified all along a certain time interval to ensure the continuity of object perception. If all these conditions are verified, then the Decision Network un-inhibit the Lexical Area and allows its activation by object representation in the Feature Network. An accurate description of this decision network can be found in [8].

 In the following each element of the lexical area will be denoted with the subscripts *i* *j* or *hk *(*i*, *h* = 1, 2,…, *M*_1_; *j*, *k* = 1,2,…, *M*_2_) and with the superscript *L*. In the present study we adopted *M*_1_ = *M*_2_ = 40. Each single element exhibits a sigmoidal relationship (with lower threshold and upper saturation) and a first-order dynamic (with a given time constant). This is described via the following differential equation:

(1) $$\tau^{L}\cdot \frac{d}{dt}x_{ij}^{L}\left(t\right)=-x_{ij}^{L}\left(t\right)+H^{L}\left(u_{ij}^{L}\left(t\right)\right),$$

where *τ* ^*L*^ is the time constant, which determines the speed of the answer to the stimulus, and *H* ^*L*^(*u* ^*L*^(*t*)) is a sigmoidal function. The latter is described by the following equation:

(2) $$H^{L}\left(u^{L}\left(t\right)\right)=\frac{1}{1+e^{-(u^{L}(t)-\vartheta^{L})\cdot p^{L}}},$$

where *ϑ* ^*L*^ defines the input value at which neuron activity is half the maximum (central point) and *p^L^* sets the slope at the central point. Equation (2) conventionally sets the maximal neuron activity at 1 (i.e., all neuron activities are normalized to the maximum).

According to the previous description, the overall input, *u*_*ij*_^*L*^(*t*), to a lexical neuron in the *i* *j* position can be computed as follows:

(3) $$u_{ij}^{L}\left(t\right)=I_{ij}^{L}\left(t\right)+V_{ij}^{F}-G^{L}\cdot \left(1-z^{L}\left(t\right)\right)-C_{ij}^{L}-I_{ij}^{\text{Bias}},$$

where *I*_*ij*_^*L*^(*t*) is the input produced by an external linguistic stimulation. *V*_*ij*_^*F*^ represents the intensity of the input due to synaptic connections from the feature network; this synaptic input is computed as follows:

(4) $$V_{ij}^{F}=\underset{h}{\sum }\underset{k}{\sum }W_{ij,hk}^{F}\cdot x_{hk},$$

where *x*_*hk*_ represents the activity of the neuron *h* *k* in the Feature Areas and *W*_*ij*,*hk*_^*F*^ the strength of synapses from the feature areas to the lexical area. The term *G* ^*L*^ · (1 − *z* ^*L*^(*t*)) accounts for the inhibition sent to the lexical area, withdrawn by the decision network. In particular, *z* ^*L*^(*t*) is a binary variable representing the output of the decision network (1 in case of correct detection, 0 in case of incorrect detection—see [8]); hence, the strength of the inhibition sent to the Lexical Area is *G* ^*L*^ when the decision network is in the OFF state and becomes 0 when the decision network shifts to the ON state. It is worth noting that the external linguistic input *I*_*ij*_^*L*^(*t*), when present, is set sufficiently high to overcome the inhibition entering into the lexical area. The term *C*_*ij*_^*L*^ (not included in the previous model versions) represents the competitive inhibition that the neuron at position *i* *j* in the lexical area receives from other words in the lexical area. This competition is triggered only in case of words representing the same object (as in bilingualism) and is computed as follows:

(5) $$C_{ij}^{L}=\underset{h}{\sum }\underset{k}{\sum }W_{ij,hk}^{I}\cdot x_{hk}^{I},$$

where *x*_*hk*_^*I*^ is the output of the inhibitory interneuron at position *h* *k*, and *W*_*ij*,*hk*_^*I*^ are the inhibitory synapses from a presynaptic inhibitory interneuron at position *hk* to the post-synaptic neuron at position *i* *j* in the lexical area. 

Finally, the term *I*_*ij*_^Bias^ in (3) represents an external inhibitory input, coming from high-level top-down influences, which try to inhibit a nontarget word. This input is normally set to zero but may assume a high value in problems like language selection or language switching (see discussion). 

The inhibitory interneuron output is computed with equations similar to those of (1) and (2), with an analogous meaning of symbols, that is,

(6) $$\tau^{I}\cdot \frac{d}{dt}x_{ij}^{I}\left(t\right)=-x_{ij}^{I}\left(t\right)+H^{I}\left(x_{ij}^{L}\left(t\right)\right),$$

with

(7) $$H^{I}\left(x_{ij}^{L}\left(t\right)\right)=\frac{1}{1+e^{-(x_{ij}^{L}(t)-\vartheta^{I})\cdot p^{I}}}.$$

It is worth noting, in (6) and (7), that the inhibitory interneuron receives excitation only from the excitatory neuron in the lexical area at the same position *i* *j*.

### 2.3. Synapse Training

Training has been subdivided in three different phases: (i) learning of objects, (ii) learning of L1 words, and (iii) learning of L2 words. The first two phases have been already described in a previous paper; hence, only some general ideas are given here. Phase (iii) is new and described in greater detail.

During the first phase, objects are individually given to the network with all their four features, and the synapses linking the feature areas are trained via a time varying Hebbian mechanism. This is described in detail in [8]. At the end of this phase, objects can be recognized even in the presence of incomplete or moderately altered inputs [8].

During the second phase, an object (already learned in phase (i)) is given to the network together with the corresponding word. Synapses linking the word and the object features (in both directions) are then learned with a Hebbian mechanism. This is described in [9]. At the end of this phase, objects can evoke the corresponding words and words can evoke the sensory-motor object representation in the feature areas. Moreover, several words and their objects representation can coexist by oscillating in time division in the gamma range.

The third phase consists in learning words of a second language. To this end, we assumed that a word in the first language (named L1 word), previously learned in phase (ii), is given to the subject together with a new word (L2 word) representing the same object in a second language. Of course the L1 word activates the object representation in the feature areas, and the synapses linking the feature areas to the L2 word (i.e., synapses *W*_*ij*,*hk*_^*F*^ in (4)) are learned with a Hebbian mechanism (similar to that used in phase (ii)). Moreover, the inhibitory synapses linking the inhibitory interneurons to the lexical area (i.e., synapses *W*_*ij*,*hk*_^*I*^ in (5)) are also learned with a Hebbian mechanism. In fact, during this phase, the L1 and the L2 words are active, and so, also the corresponding interneurons are active. The equations for synapse learning are

(8) $$\begin{array}{cc} W_{ij,hk}^{F}\left(t+T_{S}\right) & =W_{ij,hk}^{F}\left(t\right)+\beta_{ij,hk}^{F}\cdot x_{ij}^{L}\left(t\right)\cdot x_{hk}\left(t\right),\\ W_{ij,lm}^{I}\left(t+T_{S}\right) & =W_{ij,lm}^{I}\left(t\right)+\beta_{ij,hk}^{I}\cdot x_{ij}^{L}\left(t\right)\cdot x_{lm}^{I}\left(t\right), \end{array}$$

where *x*_*ij*_^*L*^(*t*), *x*_*hk*_(*t*), and *x*_*lm*_^*I*^(*t*) represent the activity of the excitatory neuron at position *i* *j* in the lexical area, the Wilson-Cowan oscillator at position *h* *k* in the feature area, and of the inhibitory interneuron at position *l* *m*, respectively, and *β* represent the learning rates. Finally, we assumed that synapses cannot overcome a maximum saturation value. This is realized assuming that the learning rates are progressively reduced to zero when synapses approach saturation.

## 3. Results

Simulations have been performed at three different moments of the second-language learning process: (i) at the beginning of training, when the second language word has never been perceived before, (ii) during an intermediate learning moment, when synapses linking the object with the L2 word are still weak, that is, much smaller than the synapses linking the same object with the L1 word, and (iii) after a long training period, when the synapses linking the object to the L2 word are almost as strong as the synapses from the same object to the L1 word. 

Three exemplary cases are presented for each training phase, characterized by different inputs to the model: L2 word as input to the model, L1 word as input to the model, and the object features as input to the model.

**Figure 2:**
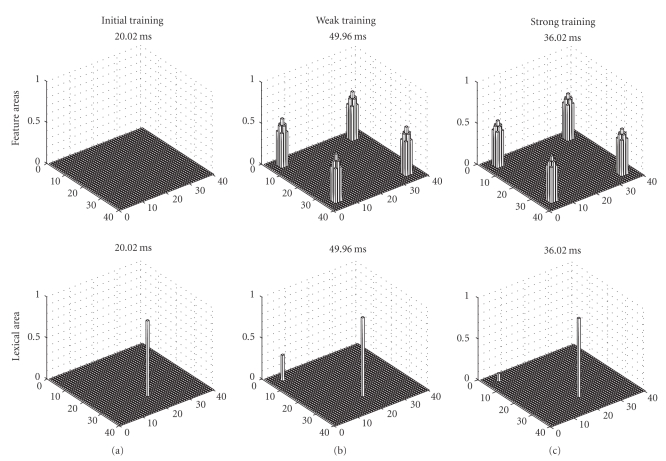
Some snapshots of the model response at some instant of the simulations, performed when the L2 word is given as input to the network. The upper panels describe activity in the feature areas, while the bottom panels represent the activity in the lexical area. The L2 word is represented by neuron activity in position 20, 30 of the lexical area, while the corresponding L1 word is represented by activity in position 5, 5. Results of three simulations are given, characterized by a different proficiency of the second language: beginning of L2 training (a), after a weak L2 training (b), and after a prolonged L2 training (c). It is worth noting that, after weak training, the L2 word can evoke the correct object representation in the feature areas (represented by simultaneous activation of four features), but it still requires activation of the L1 word. After prolonged training the L2 word becomes almost completely autonomous from L1.

Figure 2 shows the model response in the feature areas (upper panels) and in the lexical area (bottom panels) at a particular instant of the simulation, when the L2 word is used as input. In each simulation, the external input activates the corresponding neuron in the lexical area. At the beginning of training (Figure 2(a)) this word cannot evoke any object representation in the feature areas. After moderate training (Figure 2(b)) the L2 word can evoke the representation of the object in the feature areas (with the appearance of four activation bubbles, which represent the four features of the object). However, it is remarkable that also the L1 word representation is activated in the lexical area. This signifies that an L2 word is able to evoke the correct representation of the corresponding object, but it still requires some participation of the L1 word. In other terms, at this stage of training the second language still takes advantage of the first language, and both words (the L2 word and the L1 word) participate synergistically to the object representation. Conversely, after a strong training (Figure 2(c)) the L2 word can evoke the object representation with just a negligible activation of L1. This means that L2 has become almost completely independent of any L1 support.

**Figure 3:**
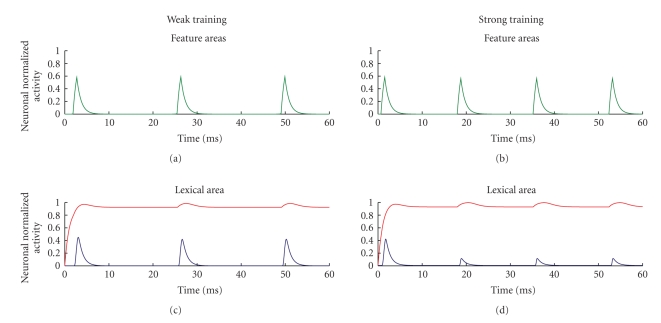
Temporal pattern of neuron activity in the feature areas representing the object (a) and (b) and in the lexical area (c) and (d) in the same simulations as in Figure 2, in case of weak training (a) and (c) and in case of strong training (b) and (d). All object features oscillate in synchronism in the gamma band (about 40 Hz). The L2 word, stimulated from an external input, exhibits quite a constant activity close to maximal saturation (red line) while the L1 word, triggered by the object representation, exhibits a significant activity in the gamma band (blue line).

The temporal patterns of neuron activities are given in Figure 3, when the network is stimulated with the L2 word as input. In case of weak training, L2 word can evoke the object representation (with the four features which oscillate in phase in the gamma band, at about 40 Hz, Figure 3(a)). However, it is noticeable the activation of the L1 word in the lexical area, which oscillates with the same phase as the object representation, Figure 3(c). After a prolonged training, the L2 word is able to almost entirely suppress activity of the L1 word, thanks to the presence of a strong competitive mechanism. The subject can use L2 without evoking L1, Figures 3(b) and 3(d).

**Figure 4:**
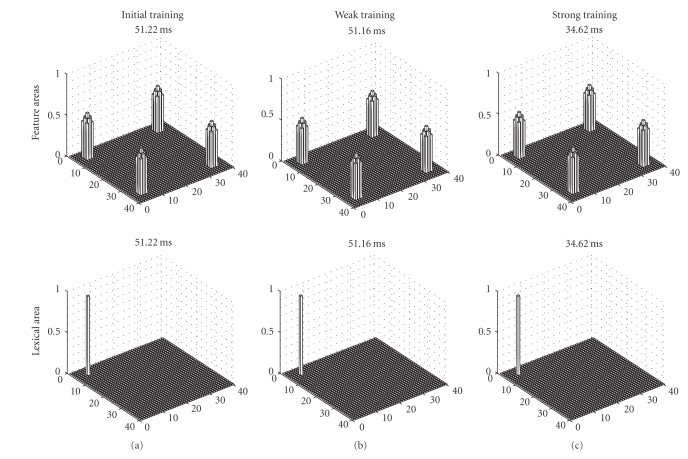
Some snapshots of the model response at some instant of the simulations, performed when the L1 word is given as input to the network. The upper panels describe activity in the feature areas, while the bottom panels represent the activity in the lexical area. The L1 word is represented by neuron activity in position 5, 5 of the lexical area, while the corresponding L2 word is represented by activity in position 20, 30. Results of three simulations are given, characterized by a different proficiency of the second language: beginning of L2 training (a), after a weak L2 training (b), and after a prolonged L2 training (c). It is worth noting that, at any training level, the L1 word can evoke the correct object representation in the feature areas (represented by simultaneous activation of four features), without any significant participation of the L2 word.

Figure 4 shows snapshots of neuron activation in the different areas of the model, when model is stimulated with the L1 word as input. Independently of the training length, the L1 word can evoke a correct object representation in the feature areas without any significant participation of L2. This signifies that, at any stage of learning, L1 is independent of the new language and, even after a prolonged second-language training, it is able to recover an object by totally inhibiting the corresponding L2 word. 

**Figure 5:**
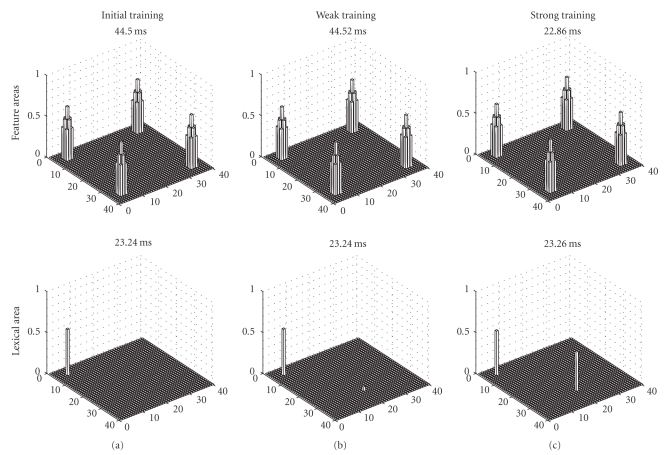
Some snapshots of the model response at some instant of the simulations, performed when the object (i.e., its four features) is given as input to the network. The upper panels describe activity in the feature areas, while the bottom panels represent the activity in the lexical area. The L2 word is represented by neuron activity in position 20, 30 of the lexical area, while the corresponding L1 word is represented by activity in position 5, 5. Results of three simulations are given, characterized by a different proficiency of the second language: beginning of L2 training (a), after a weak L2 training (b), and after a prolonged L2 training (c). It is worth noting that, at the beginning of training and after a weak training, the external object can evoke only the L1 word without any significant activity of the L2 word. Conversely, after prolonged training, the external object simultaneously evokes both the L1 word and the L2 word, despite the presence of a competitive mechanism. This requires the participation of a higher-level mechanism (perhaps inhibitory) to resolve the conflict.

The third case (Figure 5) shows model behavior when the network is stimulated with the entire object representation (i.e., all four features are given to the network as an external input). Of course, at the beginning of training the object evokes only the L1 word. Similarly, after a moderate training, when the second language is just poorly known, the object evokes the L1 word only, and the L2 word is almost completely inhibited. However, after a prolonged training, the object representation evokes both the L1 word and the L2 word, which coexist despite the presence of a reciprocal competitive inhibition. 

**Figure 6:**
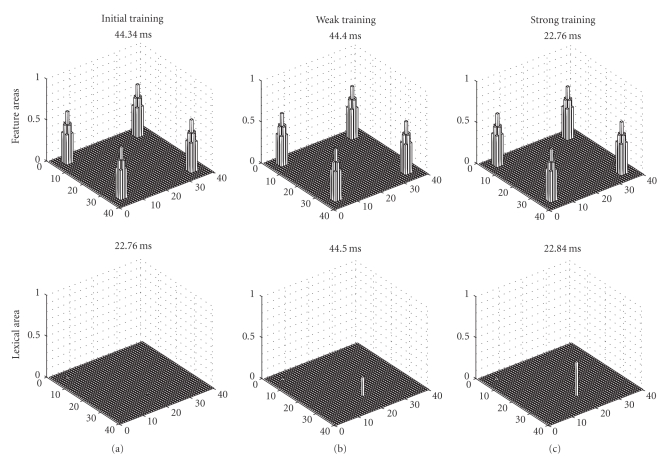
Some snapshots of the model response at some instant of the simulations, performed when the object (i.e., its four features) is given as input to the network. Simulations are the same as in Figure 5, with the same meaning of figures, but in this case we assumed that a strong top-down inhibitory input (from a higher control center) targets all L1 words. It is noticeable that, in this situation, the object representation can evoke the L2 word, but with a different level of activation depending on the proficiency level.

The previous simulations show that, if a subject has a low-proficiency L2, an external object automatically activates L1, while L2 is inhibited. Conversely, for a high-proficiency L2 subject, word production caused by an external object causes a conflict between L1 and L2 words. Some problems, thus, arise. How can a low-proficiency L2 subject produce a correct L2 word (for instance, when he/she is forced to use L2 in a foreign context or a classroom)? And how can the conflict between L1 and L2 words be solved in high-proficiency L2 subjects? To answer these questions, model must assume the presence of a further top-down inhibitory input (i.e., the term *I*_*ij*_^Bias^ in (3)) probably coming from higher cognitive centers, which is directed to the nontarget language. As an example, in Figure 6 we repeated the same simulations as in Figure 5 (object as input to the network, i.e., the word production paradigm) assuming that all L1 words are receiving an inhibitory input from an external source. Results show that, in this condition, the presentation of the object engages activation of the L2 word, less active in the low-proficiency case (Figure 6(b)) and more active in the high-proficiency case (Figure 6(c)), but without any interference from L1.

## 4. Discussion

The term “bilinguals” means people who can use two languages in their life, a first or native language usually denoted as L1 and a second language named L2. Bilingualism, of course, entails several complex problems, which are still debated in the psycholinguistic literature. A first problem is whether the second language (or L2) makes use of the same neural structures as L1 or whether different structures and different mechanisms underlie the acquisition of L2. A second fundamental aspect of bilingualism is the necessity of some control mechanisms to establish which language should be used at a given moment and in a given context and which language should be inhibited, for both what concerns word production and word comprehension. 

Most studies on bilingualism appeared in recent years (some of them summarized by Abutalebi [12]) making use of functional neuroimaging techniques (such as PET or fMRI) to detect which brain regions are involved or activated during a specific psycholinguistic test. Although these studies provide important information on brain organization in bilingualism, they do not explore the neural mechanisms involved. Moreover, language is a typical characteristic of humans; hence, animal experiments cannot be used to deepen our knowledge of the problem.

In this situation, mathematical models and computer simulations, although drastically simplified compared with the reality, may provide important contributions to clarify the possible mechanisms involved in language processing (at least for what concerns basic aspects as word recognition and word production) and to convert current hypotheses into rigorous quantitative theories. Indeed, several current theories on language frequently use expressions like “competition”, “inhibition”, “neural activity”, and “control” and can be regarded as “qualitative models”, which may certainly benefit of a more accurate quantitative formalization.

The model presented in this work whishes to represent a first step in that direction. However, it aspires to describe only the lexical-semantic aspects of language, without any inclusion of grammatical issues: in particular, attention is focused on *word recognition* (i.e., the process through which a word is converted into a coherent object representation) and *word production* (the process through which the representation of an object is converted into a word). 

The main assumption of the model is that neurons labeling words, and neurons describing their semantic object representation over different areas, are linked together via excitatory synapses. It is remarkable that the model, for the sake of simplicity, does not incorporate the two “external” aspects of this processing stream, namely, how phonemes are converted to lemmas (and words) and how the sensory-motor information coming from senses is generalized to arrive at an abstract representation of objects. 

A second fundamental aspect of the model is that the synaptic links between object representation and words are learned during a training phase, in which the object and the corresponding word are presented together. This particularly of the model allows learning of L2 words with the same basic mechanism as that of L1, supposing a training phase in which the L2 word is presented together with a previously learned L1 word. 

The latter assumption, which is fundamental in our work, received several confirms from recent neuroimaging studies (some of them summarized by Perani and Abutalebi [13] and Abutalebi [12]). Indeed, a traditional viewpoint in the neurolinguistic literature, which dominated for more than one century, was that the first and second languages depend on different cerebral structures and on different neural mechanisms [14]. Following this line of thinking, a recent qualitative model (named the Ullman declarative/procedural model [15]) assumes that processing of L2, acquired late on life, depends upon different cognitive mechanisms and different cerebral structures than L1. Several recent results, however, using functional neuroimaging data [16, 17], contradict this hypothesis for what concerns the lexical-semantic aspects of L2 (although structural differences between L1 and L2 may effectively occur for what concerns the grammatical aspects). As summarized in the review paper by Abutalebi [12] “The emerging picture from studies investigating the lexical-semantic domain is that L2 is essentially processed through the same neural networks underlying L1 processing.”

Assuming that the lexical-semantic aspects of L2 are acquired through the same neural structures and the same plasticity rules as L1, the present model makes some testable predictions, at different stages of the learning process, which can be compared with present functional neuroimaging data and with present theories on bilingualism. In the following, these predictions will be separately discussed for what concerns the neural representation of L2 (i.e., the neural substrates of words and concepts) and the control mechanisms implicated in bilingualism.

*The neural representation of L2*—An interesting result of our simulations is that, at a low proficiency level, when L2 is just poorly learned, the recognition of an object from an L2 word implicates the participation of L1. This is evident in Figures 2 and 3 by the activation of the L1 word in the lexical area. These simulation results agree with some psycholinguistic theories. For instance, Kroll and Stewart [18] state that, during the early stages of L2 acquisition, L2 depends on L1 to access meaning for its lexical items. A consequence of this idea is that, at low proficiency level, the use of an L2 word causes a greater neural activation in the lexical and in the inhibitory areas, compared with the use of L1 words. Let us consider the situation depicted in Figure 2(b), and in Figures 3(a) and 3(c): here one can observe two zones of the lexical area which are simultaneously active; of course, the inhibitory interneurons are also active in same zones (since they receive their input directly from the lexical area). This signifies that a greater activation is recruited when the low-proficiency subject is trying to use an L2 word. This result is supported by neuroimaging data, although it is quite difficult to force this parallelism beyond a very qualitative level. Studies investigating the lexical-semantic domain show that bilinguals with low-proficiency L2 entail additional brain activity compared with the L1 word or compared with monolingual subjects: the increased activity is especially observed in the left inferior frontal gyrus and in prefrontal areas [16, 19]. 

In addition, model predicts that, with an increase in L2 proficiency, the neural activity engaged by L2 words is reduced and becomes progressively comparable with that engaged by L1 words. This model predictions reflect the so-called Green's convergence hypothesis [20], according to which higher levels of proficiency in L2 produce a lexical-semantic representation which more closely resembles that produced by L1. Indeed, neuroimaging studies have reported similar activations in the left frontal and tempo-parietal brain areas when a subject performs a word production task, in case of L1 and high-proficiency L2 [21, 22]. This signifies that highly proficient bilinguals do not need to recruit additional resources when using L2 to achieve similar results as L1. 

*Control mechanisms*—The model stresses the need of some control mechanisms, which must be put into action to solve several conflicts involved in bilingualism: which language must be used at a given moment and in a given context? How can a low-proficiency subject use L2 words avoiding any interference from the stronger L1? A common hypothesis in the neurolinguistic literature assumes that control entails a sort of competition between the two languages and that this competition is solved by inhibiting the nontarget language [23]. Of course, competition can occur both at the level of phonemes (for instance, the English word “dog” competes with the English word “dot”), and at a semantic level (the English word “dog” competes with the French word “chien”). Since the first aspect, linking phonemes to words, is not considered in this model, only the second kind of competition will be considered below.

According to a former assumption by Rodriguez-Fornells et al. [24], model suggests the presence of two distinct but interrelated control mechanisms, which work through inhibition. 

A local bottom-up inhibitory mechanism aims to inhibit the weaker language without the participation of higher control centers. This mechanism is directly implemented within the model via inhibitory interneurons and is trained with the same Hebbian rule used to link words and objects. In the present simulations, this mechanism operates when L2 proficiency is lower than L1 proficiency and the subject perceives an object and must produce the corresponding word. In this condition, word production in response to an object spontaneously engages L1 without any activation of the L2 word (L2 is inhibited by the internal competition; see Figure 5 central column). 

Results in Figures 5 and 6 stress the need for a second top-down control system, which can be considered an external input to the model and inhibits one language to favor the other. This situation may involve paradigms like language switching, language translation, or language selection. In our simulations this external input may become necessary when two languages have a similar proficiency level (compare Figures 5(c) and 6(c)) or when the subject is forced to use a low-proficiency language despite the interference from the high-proficiency one (compare Figures 5(b) and 6(b)). A classic point of view is that these conflicts are solved by a dynamical inhibitory input to the nontarget language, and this may originate from various brain areas classically related to cognitive control, such as the caudate nucleus, the prefrontal cortex, and the anterior cingulate cortex [25–27]. An interesting question is whether this top-down control system is specifically dedicated to language or represents a more general structure, devoted to conflict resolution independently of its explicit domain. Inclusion of such an external mechanism may be the subject of future improvement of the present model. 

In conclusion, the present work represents a first attempt to study lexical-semantic aspects of language, such as word production and word comprehension, using neural networks and computer simulations. Assuming that the same neural structures and the same learning mechanisms operate for L1 and L2, the model makes several predictions which agree with some psycholinguistic theories and recent neuroimaging data. Further aspects which require model extension are especially concerned with language control via top-down inhibitory mechanisms. Introduction of this mechanism may allow more complex paradigms to be simulated, such as language selection, language switching, or language translation.
